# Machine Learning for Coronary Plaque Characterization: A Multimodal Review of OCT, IVUS, and CCTA

**DOI:** 10.3390/diagnostics15141822

**Published:** 2025-07-19

**Authors:** Alessandro Pinna, Alberto Boi, Lorenzo Mannelli, Antonella Balestrieri, Roberto Sanfilippo, Jasjit Suri, Luca Saba

**Affiliations:** 1 Department of Radiology, University of Cagliari, 09124 Cagliari, Italy; alessandpi3@gmail.com (A.P.);; 2 Department of Cardiology, Azienda Ospedaliera G. Brotzu, 09124 Cagliari, Italy; 3 Department of Radiology, IRCCS Istituto Auxologico Italiano, San Luca Hospital, 20149 Milan, Italy; 4 Department of Vascular Surgery, University of Cagliari, 09124 Cagliari, Italy; 5 Stroke Monitoring and Diagnostic Division, AtheroPoint™, Roseville, CA 95661, USA; 6 Global Biomedical Technologies, Inc., Roseville, CA 95661, USA; 7 Department of Electrical and Computer Engineering, Idaho State University, Pocatello, ID 83209, USA

**Keywords:** ML, AI, coronary plaque, OCT, IVUS, CCTA, vulnerable plaque, DL, multimodal imaging

## Abstract

Coronary plaque vulnerability, more than luminal stenosis, drives acute coronary syndromes. Optical coherence tomography (OCT), intravascular ultrasound (IVUS), and coronary computed tomography angiography (CCTA) visualize plaque morphology in vivo, but manual interpretation is time-consuming and operator-dependent. We performed a narrative literature survey of artificial intelligence (AI) applications—focusing on machine learning (ML) architectures—for automated coronary plaque segmentation and risk characterization across OCT, IVUS, and CCTA. Recent ML models achieve expert-level lumen and plaque segmentation, reliably detecting features linked to vulnerability such as a lipid-rich necrotic core, calcification, positive remodelling, and a napkin-ring sign. Integrative radiomic and multimodal frameworks further improve prognostic stratification for major adverse cardiac events. Nonetheless, progress is constrained by small, single-centre datasets, heterogeneous validation metrics, and limited model interpretability. AI-enhanced plaque assessment offers rapid, reproducible, and comprehensive coronary imaging analysis. Future work should prioritize large multicentre repositories, explainable architectures, and prospective outcome-oriented validation to enable routine clinical adoption.

## 1. Introduction

Ischemic heart disease (IHD) caused by atherosclerosis is a leading cause of cardiovascular death globally. According to data from the Global Burden of Disease Study [1], this condition caused about 9.44 million deaths in 2021, with a total disease burden expressed in years of life lost due to disability (DALYs) of 185 million. A systematic review with meta-analysis encompassing 14 studies and 37,808 participants revealed that even among people with coronary artery calcium scores (CACSs) of zero, the prevalence of non-calcified coronary plaques was approximately 10% [2]. In recent years, it has been demonstrated that the degree of stenosis is not the only parameter to stratify the risk of events related to the atherosclerotic pathology of the coronary artery and features of high-risk plaques (HRPs) can predict major adverse cardiovascular events (MACEs) [3,4,5]. HRP features, such as low-attenuation plaque, a napkin-ring sign, spotty calcification, and positive remodelling, are significantly correlated with heightened MACE risk [4], with the presence of coexisting HRP features exacerbating risk [3,4]. In this scenario, coronary computed tomography angiography (CCTA) and intravascular imaging techniques—such as intravascular ultrasound (IVUS) and optical coherence tomography (OCT)—have demonstrated their effectiveness in evaluating and delineating plaque characteristics that operate as indicators of impending cardiovascular incidents, guiding patient classification and treatment [6,7,8]. Artificial intelligence (AI) comprises many computer science methodologies that empower machines to execute tasks comparable to human intelligence. In the domain of cardiovascular imaging, AI has experienced considerable improvements and progress in recent years [9,10,11,12]. AI models have demonstrated their effectiveness in various coronary imaging domains, including the evaluation of plaque form and composition [13,14,15,16]. The purpose of this review is to explore the principles concerning AI applied to coronary plaque characterization by exploring the literature on AI-based models in CCTA, IVUS, and OCT for the segmentation and HRP features detection. The benefits and drawbacks of different AI models will also be analyzed.

## 2. Materials and Methods

A narrative exploration of the literature was performed in PubMed, Scopus, Embase, and Google Scholar, covering the period 1 January 2019–31 March 2025. The complete search strings for each database are provided in the Supplementary Materials (Supplementary Table S1).

Articles were included if they reported quantitative performance metrics on machine learning or artificial intelligence applications for coronary plaque characterization using at least one of OCT, IVUS, or CCTA. Studies were excluded if they were conference proceedings, non-peer-reviewed material, non-English publications, studies without quantitative ML/AI methods, and animal or phantom studies without human validation. Titles and abstracts were screened for methodological and clinical relevance, with inclusion decisions jointly agreed upon by all authors.

Beyond the primary database search, additional relevant studies were identified through citation tracking of key articles and reference lists of included publications.

The quality and relevance of included studies were assessed based on sample size adequacy, validation methodology, and clarity of reported performance metrics. The final selection comprised 27 studies that formed the basis of the qualitative synthesis. A narrative design was chosen because the field is highly heterogeneous in terms of imaging protocols, machine learning architectures, and evaluation metrics, making the goal of mapping key technical trends more appropriate than quantitative pooling of results.

## 3. Coronary Artery Disease

### 3.1. Pathophysiology

Coronary atherosclerosis is a multifaceted inflammatory condition marked by the deposition of lipids, fibrous components, and calcification within artery walls [17,18]. Pathogenesis encompasses endothelial dysfunction, inflammatory mechanisms, and oxidative stress [19], and the process of plaque formation and progression is influenced by both hereditary and environmental factors [20]. Historically regarded as a cholesterol storage disorder, recent studies highlight the significance of multiple factors in the process of atherogenesis, such as inflammation [18] and wall shear stress [21]. Neovascularization in coronary artery atherosclerotic plaques elevates the risk of rupture and bleeding [22], with inflammation and intraplaque hemorrhage common in the proximal areas of ruptured plaques [23], underscoring the significance of angiogenesis in plaque formation. Furthermore, positive remodelling correlates with an elevated risk of plaque rupture, as they have a higher probability of presenting HRP features [24,25].

The primary causes of acute coronary syndrome (ACS) are plaque rupture and subsequent thrombosis [26]; indeed, the composition and susceptibility of plaques, rather than the degree of stenosis, are critical drivers of cardiovascular events [27]. Although plaque rupture is the primary cause of ACS, plaque erosion—defined by endothelial denudation without fibrous cap disruption—constitutes a notable alternative mechanism, responsible for approximately 1/3 of acute coronary events, especially in younger patients [28].

### 3.2. Pathological Anatomy

The anatomopathological classification of coronary atherosclerotic lesions has evolved over time. Currently, two main systems are used for classification: the American Heart Association (AHA) classification, proposed by Stary et al. [29], which focuses primarily on plaque development from initial fatty streaks to advanced lesions with lipid cores, and the same classification, modified by Virmani et al. [30], which places greater emphasis on plaque morphology and vulnerability. Both classifications, along with their respective characteristics and differences, are detailed in Table 1.

Atherosclerosis development typically adheres to a sequence of morphological alterations shown below:

- Fatty streaks: Fatty streaks are formations that typically merge into elongated lesions measuring 1 cm or greater in length. Their composition consists of lipid-filled foamy macrophages, and morphologically, they are minimally raised; therefore, they do not induce substantial hemodynamic alterations [31].
- Atherosclerotic plaque: Intimal thickening and lipid accumulation characterize atherosclerotic plaques [32]. Atherosclerotic plaques are raised, yellow-white lesions ranging from 0.3 to 1.5 cm in diameter. They can coagulate to form larger masses. These tend to be focal lesions, and this characteristic can be explained by local flow irregularities, such as turbulence at the bifurcation points, at the preferred site of plaque formation [33,34]. Atherosclerotic lesions exhibit a structural organization with three primary compositional elements: first, cellular elements such as smooth muscle cells (SMCs), macrophages, and T lymphocytes; second, the extracellular matrix (ECM), consisting of collagen fibres, elastin, and various proteoglycans that ensure structural integrity; and third, lipid accumulations, both in intracellular and extracellular compartments [35]. The structural organization typically shows a superficial fibrous cap characterized by a predominance of SMCs embedded in dense collagenous tissue. Deep within this superficial fibrous cap is a necrotic core containing lipids, cellular debris, foamy cells (macrophages and SMCs embedded in lipids), fibrin deposits, and thrombotic material in different stages of organization. Peripherally, areas of neoangiogenesis are observed, which contribute to plaque growth and instability. Atheromas in advanced stages tend to undergo calcification processes [30,36].

## 4. Notions of AI

AI refers to methods that enable machines to learn from experience and replicate human cognition [10,12]. At its core lies machine learning (ML), the computer statistical discipline whose algorithms improve as data accumulate [37,38]. Deep learning (DL)—an ML subset—uses deep neural networks (DNNs) to process vast datasets without explicit instructions. A DNN consists of successive input, hidden, and output layers of interconnected neurons. During training, connections leading to correct outputs are strengthened, while those producing errors are weakened. Through this iterative adjustment, the network refines itself until the desired accuracy or performance is reached, enabling DNNs to replace rule-based techniques by extracting features autonomously.

Beyond these core definitions, practical deployment in medical imaging increasingly leverages transfer learning—re-using weights pre-trained on millions of natural images—together with hybrid supervision schemes (semi-, weakly and self-supervised learning) that reduce annotation burden [38,39]. State-of-the-art pipelines now embed explainability modules (e.g., saliency maps and attention roll-outs) to meet regulatory demands for transparent decision-making, while uncertainty estimation quantifies model confidence. These refinements, combined with domain-specific data augmentation, have made ML/DL the de facto standard for automated coronary plaque analysis.

**Figure 1:**
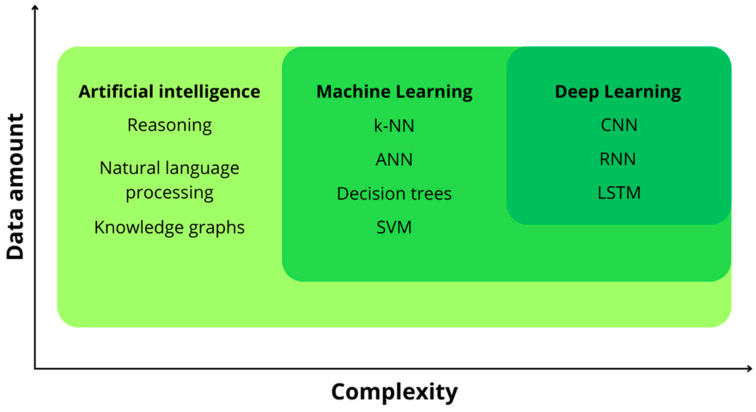
The nested subsets within the AI domain are depicted in the diagram, showcasing distinct algorithmic instances in each category. The axes show that as the requirements for data amount and computational complexity increase, so does the progression from AI to DL. k-NN: k-nearest neighbours; ANN: artificial neural network; SVM: support vector machine; CNN: convolutional neural network; RNN: recurrent neural network; LSTM: long short-term memory network.

Under this broader framework, Figure 1 shows the hierarchical relationship among AI, ML, and DL, highlighting how progression across these domains is accompanied by growing data requirements and algorithmic complexity [38].

**Figure 2:**
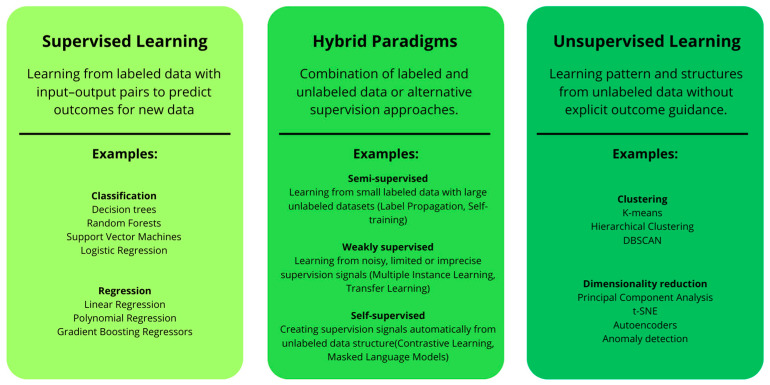
Taxonomy of machine learning tasks categorized by supervision type, illustrating the spectrum from supervised learning (fully labelled data) through hybrid paradigms (semi-, weakly, and self-supervised learning) to unsupervised learning (no labelled data), with representative algorithms for each category. DBSCAN: Density-Based Spatial Clustering of Applications with Noise; t-SNE: t-distributed Stochastic Neighbour Embedding.

ML algorithms themselves can be grouped according to the extent and reliability of the labelled information available during training: Fully labelled datasets produce supervised learning; the absence of labels defines unsupervised learning; and intermediate or imperfect annotations produce hybrid paradigms. The latter encompasses semi-supervised, weakly supervised, and self-supervised learning, each differing in the proportion or quality of annotated data they exploit [39]. This taxonomy is presented in Figure 2, which concisely summarizes the distinctive characteristics and corresponding methods related to each supervision paradigm.

The selection of a learning architecture involves trade-offs between data demands, interpretability, and cross-domain adaptability (see Table 2 for a concise comparison). Conventional machine learning algorithms (e.g., Random Forest and support vector machine) are lightweight and interpretable, perform well on small, well-engineered feature sets, but plateau on complex imaging data. Convolutional neural networks (CNNs) overcome this ceiling by learning hierarchical visual features end-to-end and now deliver expert-level segmentation and plaque classification at the cost of large labelled datasets and GPU-level computation. Furthermore, 3D-CNNs and encoder–decoder variants boost spatial consistency along OCT pullbacks but further increase memory footprint. Transformer architectures capture long-range context and improve erosion detection on OCT (AUC 0.94) yet remain data-hungry and computationally intense. Generative adversarial networks (GANs) are valuable for data augmentation and real-time IVUS segmentation but suffer from training instability and mode collapse. Finally, ensemble and hybrid approaches enhance robustness across centres, although their complexity complicates deployment on point-of-care hardware.

## 5. High-Risk Plaque (HRP) Features

HRP represent the fundamental pathological substrate in the development of ACS, and the use of various non-invasive (CCTA) and invasive (OCT and IVUS) imaging techniques is essential for their identification. Their identification by these different techniques is becoming increasingly important in the prognostic stratification of patients with CAD [40,41]. Histologically, vulnerable plaques are characterized by a large necrotic lipid core, inflammation, thinned fibrous cap, neovascularization, and arterial remodelling. These different features (summarized in Table 3) can be detected in various ways: with CCTA, mainly napkin-ring signs, low-attenuation plaques, positive remodelling, and punctiform calcifications are observed; with IVUS, necrotic core, positive remodelling, and distribution of calcifications are evident; and with OCT, due to its high resolution, thin fibrous caps, erosions, macrophage infiltrates, and neovascularization are identified.

**Figure 3:**
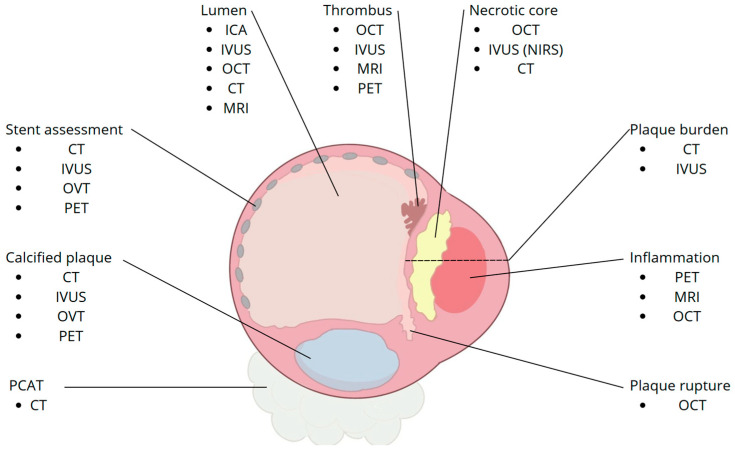
Schematic cross-section of a coronary artery highlighting the imaging modalities best suited for assessing key plaque features. ICA: invasive coronary angiography; IVUS: intravascular ultrasound; OCT: optical coherence tomography; CT: computed tomography angiography; MRI: magnetic resonance imaging; PET: positron emission tomography; NIRS: near-infrared spectroscopy; PCAT: pericoronary adipose tissue.

**Figure 4:**
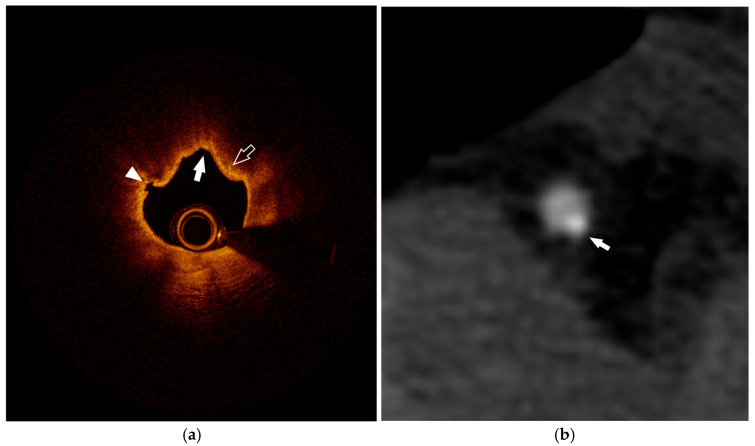
Multimodality imaging for plaque assessment. (**a**) right coronary artery OCT image showing a thin-cap fibroatheroma (TCFA) with minimal lumen area < 3.5 mm^2^, extensive lipid-arc from 7 to 2 o’clock (solid arrow), and presence of bright spots (open arrow) consistent with macrophage infiltration due to plaque inflammation; at 9 o’clock, endothelial disruption is observed (arrowhead). (**b**) right coronary artery CCTA image demonstrating an eccentric calcified atherosclerotic plaque at 5 o’clock (white arrow).

The inherent limitations of each technique can be overcome through the combined and integrated use of these three modalities, which, with good reason, should be considered as complementary techniques. Figure 3 provides a schematic cross-sectional overview of the main high-risk plaque components and the imaging modalities most suitable for their assessment, while Figure 4 shows examples of multimodality imaging features.

### 5.1. OCT

Among those listed, OCT represents the technique with the highest spatial resolution (10–20 μm), which allows accurate and precise assessment of plaque morphologic features, with a focus on surface features [40]. It excels in the identification of thin fibrous caps, for which it allows the accurate and reproducible thickness measurement, with a detection limit of less than 75 μm, and offers unique capabilities for the assessment of vulnerability features such as macrophage infiltration, microchannels, and the presence of thrombi; it has also proven particularly effective in the characterization of the lipid core and the detection of surface calcifications. However, due to its inherent technical characteristics, it has limitations in terms of tissue penetration (limited to 1–2 mm), narrow field of view, and presence of shadowing artifacts. Prati et al. [42] proposed an OCT-based vulnerability grading system that includes minimum luminal area < 4 mm^2^, cap thickness < 75 μm, and presence of macrophages, demonstrating high accuracy in identifying culprit lesions in ACS.

### 5.2. IVUS

IVUS has an intermediate resolution (100–200 μm), which allows for greater tissue penetration, ideal for assessing deeper components of plaque and vascular remodelling [40]. It demonstrates superiority in quantifying plaque burden and detecting positive remodelling; the latter is a feature that has been strongly associated with plaque vulnerability. In addition, with advanced techniques such as virtual histology (VH-IVUS), it offers fair accuracy in tissue characterization, allowing fibrous, lipid, and calcific components to be distinguished [43]. Raffel et al. [44] demonstrated that positive remodelling detected in IVUS is significantly associated with the presence of vulnerability features identified in OCT. The main limitations of IVUS include the inability to accurately measure thin fibrous caps, the reduced ability to detect macrophage infiltration and microchannels, and the presence of acoustic shadowing that can mask structures behind dense calcifications, as well as having lower accuracy than OCT in detecting thrombus.

### 5.3. CCTA

Among the imaging techniques reviewed, CT is the only non-invasive one. It, at least as far as conventional CT is concerned, has a significantly lower resolution (about 1 mm), but has the significant advantage of being able to assess the entire coronary tree [40]. As reported by CAD-RADS 2.0 [45], CT has the ability to indirectly detect vulnerability features through surrogate markers, such as napkin-ring signs, which are indicative of thin-cap fibroatheroma (TCFA), low attenuation plaques (indicative of lipid content), punctiform calcifications, and positive remodelling. Nakazato et al. [41] reported that positive remodelling and low-attenuation plaques detected in CT were strongly associated with TCFA and macrophage infiltration identified in OCT, with odds ratios of 16.9 and 11.2, respectively. Recent advances such as radiomic analysis have significantly improved the diagnostic capabilities of CT, with Kolossváry et al. [46] demonstrating superior performance compared to conventional assessments in identifying vulnerability features, achieving area under the curve (AUC) values as high as 0.87 for NaF18 positivity, 0.80 for TCFA in OCT, and 0.72 for attenuated plaques in IVUS. Major limitations include the indirect assessment of the fibrous cap and the inability to directly detect macrophage infiltration.

## 6. Machine Learning Models for Automatic Coronary Arteries Segmentation

ML methods—especially DL techniques—have brought notable developments in the field of coronary artery segmentation. Among these, convolutional neural networks (CNNs) and their variants have become rather popular since they provide significant accuracy, repeatability, and efficiency over conventional manual or semi-automatic segmentation. These models seem to help evaluate stenosis and lower intra- and inter-observer variability, optimizing the clinical process. The main results obtained from the analyzed methods are summarized in Table 4.

### 6.1. OCT

Because of limited tissue penetration, shadow artifact presence, and variability brought about by catheter placement and blood clearance, OCT imaging presents special difficulties for automatic segmentation. In this regard, ML and DL models, especially, have shown great promise since they offer strong and accurate segmentation performance. Recent advances have investigated advanced architectures, including spatial and temporal information from sequential OCT pullbacks outside of conventional CNN frameworks. For instance, Li et al. [48] proposed spatial–temporal encoder–decoder networks to use the continuity between consecutive frames, improving segmentation consistency and lowering frame-to-frame variability. By using the naturally three-dimensional character of intravascular datasets, such techniques solve one of the main constraints of frame-based segmentation techniques, achieving F1-scores up to 0.883 for calcification segmentation, and significantly improving the Dice coefficient compared to previous 2D and 3D CNN methods. Combining a 3D CNN for identifying significant calcification lesions and a SegNet model with Tversky loss for segmentation, Lee et al. [49] created a two-stage DL approach with a sensitivity of 97.7%, specificity of 87.7%, and F1-score of 0.922 in lesion detection, showing great improvements compared to the standard one-step method (sensitivity from 77.5% to 86.2% and F1-score from 0.749 to 0.781). Using expert manual segmentation as the gold standard, Zhang et al. [47] devised two automatic methods for coronary plaque segmentation in OCT images, obtaining a classification accuracy of 95.8% with the CNN-based model and 71.9% with the SVM-based model.

### 6.2. IVUS

Recent developments in ML have greatly improved the automatic segmentation of IVUS images, addressing long-standing limits of manual and semi-automated approaches in terms of repeatability and efficiency. Matsumura et al. [50] developed a U-Net-based model for high-definition (60 MHz) IVUS, with a 92.4% agreement rate in balloon sizing when both lumen and vessel diameters were considered, and correlation coefficients of 0.992 and 0.993 for lumen and vessel areas, respectively, were achieved. Using a Gradient Boosting framework, Cui et al. [51] proposed a supervised ML method reporting a Jaccard similarity index of 96.8% and a mean error distance of 0.55 mm, outperforming more complex DL methods. Using a Pix2Pix generative adversarial network (GAN) with the ResNet backbone, Bajaj et al. [52] presented a real-time DL segmentation method showing mean differences ≤ 0.23 mm^2^ compared to expert annotations and Dice similarity coefficients up to 0.98.

### 6.3. CCTA

The complex and varied vessel morphology, low contrast with adjacent structures, and the presence of noise and artifacts make the automatic segmentation of coronary arteries from CCTA images a particularly challenging task.

By using hierarchical spatial features and preserving continuity across volumetric datasets, DL models—especially U-Net-based architectures—have notably raised segmentation accuracy in recent years. Nannini et al. [55] used a dual-stage approach comprising a 2.5D U-Net and a multichannel 3D U-Net to produce a DSC of 0.895 and a mean surface distance of 1.027 mm. This architecture showed particular strength in segmenting stenotic regions and accurately tracking vessel continuity across distal branches. High-performing models incorporating attention mechanisms and multi-path feature enhancement have also been proposed for this task. PlaqueNet, which combines a DASPP-BICECA module with deepwise residual blocks, outperformed three benchmark models (FCN, Deeplabv3, and Deeplabv3plus), achieving a Dice of 93.26%, a mean Dice of 96.63%, and a mean IoU of 93.68% [56]. By tackling issues of vessel size variation and overlapping anatomical features, a feature fusion and rectification 3D U-Net architecture has also shown enhancements in vessel boundary definition and segmentation accuracy [53]. Although exact Dice values were not reported, the architecture showed performance superior to baseline U-Net3D, with enhancements attributed to its AGFF, SAFE, and MSFA modules, which effectively combined low-level detail with high-level semantic information. Complementing these supervised approaches, Serrano-Antón et al. [54] introduced an unsupervised Ward-clustering strategy (3Axis) that achieved DSCs of 0.88 on a 10-patient test set and 0.83 in lesion-focused cases, demonstrating performance comparable to state-of-the-art CNNs while eliminating the need for manual annotations.

## 7. Machine Learning Models for Automatic Detection of Coronary Artery Plaque Vulnerability Features

Recent advancements in ML techniques have considerably improved the automatic identification of coronary artery plaque vulnerability features. Traditional manual or semi-automated methods, while widely validated, often encounter challenges related to observer dependence, reproducibility, and extensive analysis time. In response, diverse ML methods—especially CNNs and advanced variants such as Residual Networks (ResNet), generative adversarial networks (GANs), and transformer models—have demonstrated superior performance in plaque characterization tasks, exhibiting higher accuracy, sensitivity, and specificity compared to conventional techniques. Furthermore, hybrid and ensemble ML methodologies have emerged, demonstrating improved reliability and broader applicability in clinical settings. The main results obtained from the analyzed methods are summarized in Table 5.

### 7.1. OCT

The application of DL to OCT image analysis has led to significant progress in the automated identification of plaque vulnerability features, particularly in the classification and quantification of coronary calcification and lipid deposits. Among the most often used models are 3D CNNs, spatial–temporal encoder–decoder architectures, transformers, and fully convolutional networks—each meant to use contextual information from sequential OCT frames. He et al. [57] proposed a ResNet-3D-based model optimized via transfer learning and majority voting in the domain of calcified plaque classification. With an F1-score of up to 96% for binary classification of calcified against non-calcified plaques, the model exceeded equivalent 2D models and showed enhanced spatial continuity along pullbacks. Trained on a dataset comprising over 13,800 OCT images, Li et al. [48] addressed the automatic segmentation of coronary calcification using a spatial–temporal encoder–decoder network combined with DenseNet. Their use of focused data augmentation raised the Dice coefficient from 0.615 ± 0.332 to 0.756 ± 0.222, approaching the level of inter-observer human agreement. Furthermore, region-based classification reached an F1-score of 0.883 ± 0.008 and a precision of 0.964 ± 0.002, reflecting some of the highest recorded accuracies in the literature for this task. DL has also been investigated for the detection of plaque erosion. Park et al. [58] created a transformer-based model intended especially to incorporate sequential frame information. With an AUC of 0.94 at the frame level and 0.91 at the lesion level, the transformer demonstrated noticeably better performance than standard CNNs when compared to 0.85 and 0.84, respectively, with a conventional CNN architecture. Recently, the characterization of lipid plaque has also seen developments. Finally, achieving concordance levels between 88% and 93% depending on the comparison modality, Huang et al. [59] showed that calcified plaque classification results from OCT-based DL were consistent with optical properties, IVUS virtual histology, and IVUS echogenicity. Their multimodal co-registration study increases the clinical credibility of automated methods in cross-modality validation contexts.

### 7.2. IVUS

Although the current literature offers limited direct evidence on ML models specifically trained to identify histologically defined HRP features using IVUS, valuable insights can still be derived from models developed for the automatic detection of structural plaque characteristics, such as calcification and ultrasound attenuation. These features, while not definitive markers of vulnerability, have been consistently associated with adverse procedural outcomes and are often used as surrogate indicators of plaque instability in clinical practice. The application of DL models to IVUS imaging has recently demonstrated significant potential in the automated detection of coronary plaque vulnerability features, particularly in identifying attenuated and calcified plaques. Cho et al. [60] developed an EfficientNet-based DL model trained on 113,746 IVUS frames derived from 498 coronary arteries, using circumferential annotations at 0.4 mm intervals and 1° angular resolution across 360° of the vessel wall. In the angle-level evaluation, the ensemble model achieved an overall classification accuracy of 98% across three classes (attenuation, calcification, and none), with Dice similarity coefficients of 0.74 for attenuation and 0.79 for calcification. At the frame level, the ensemble model reached a sensitivity and specificity of 80% and 96% for attenuation detection, and 86% and 97% for calcification, with strong correlations to manual measurements (r = 0.89–0.95). An additional approach proposed by Li et al. [61] employed a two-stage architecture composed of three modified U-Nets to segment the media–adventitia border, the lumen, and calcified tissue. The model demonstrated high segmentation accuracy for vascular structures (DSC > 0.93), while the detection of calcified tissue yielded the best performance when using focal loss (DSC = 0.67; precision = 0.77; sensitivity = 0.72). The precision of calcification detection increased proportionally with the percentage of calcified tissue within the plaque, suggesting that higher calcium burden enhances model discrimination. Compared to commercial software (e.g., VH-IVUS), the model exhibited superior robustness in the presence of shadow artifacts and side branches, maintaining accuracy even under challenging imaging conditions.

### 7.3. CCTA

CCTA has emerged as a non-invasive and widely accessible modality for the assessment of coronary artery disease (CAD), allowing both anatomical evaluation and identification of HRP features. Recent advances in ML have significantly enhanced CCTA’s capacity to detect stenosis and vulnerable plaques. In a prospective study covering 1013 vessels, Yang et al. [66] showed that possessing ≥4 of 6 ML-selected CCTA features—MLA, lesion PAV, lipid core volume, plaque volume, proximal LAD site, remodelling index—raised five-year VOCO risk five-fold (HR 5.43), independent of stenosis and FFR. Kim [63] proposed a 2D nnU-Net-based segmentation model trained on 2978 CCTA scans to evaluate stenosis, calcification, and vulnerable plaques. The model yielded a strong correlation (r = 0.715) between the quantitative stenosis index (QSI) and invasive quantitative coronary angiography. The sensitivity and specificity for identifying vulnerable plaque were 0.929 and 0.910, respectively, indicating excellent diagnostic performance in a screening setting. Radiomics-based approaches have also shown promise in enhancing plaque characterization. Chen et al. [62] introduced a radiomic signature (RS) derived from 16 features (including shape and texture) to identify plaques considered vulnerable by IVUS. This RS demonstrated robust predictive performance (AUC = 0.77–0.81 across datasets) and was independently associated with MACEs (hazard ratio = 2.01; *p* = 0.005) during a 3-year follow-up, surpassing conventional anatomic plaque metrics. Moreover, Buckler et al. [64] validated an ML classifier trained with histology-confirmed tissue labels to identify plaque stability phenotypes (minimal, stable, and unstable). Their model showed excellent agreement with histopathological ground truth (κ = 0.82; AUC = 0.97 for unstable plaque), outperforming traditional diameter stenosis metrics, which showed weak correlation with plaque vulnerability (κ = 0.25). Finally, Al’Aref et al. [65] employed a boosted ensemble ML model (XGBoost) within the ICONIC study to predict culprit lesions among patients with ACS, using lesion-level quantitative features extracted from baseline CCTA. The model outperformed traditional stenosis-based metrics (AUC = 0.774 vs. 0.599) in identifying lesion precursors of ACS events, demonstrating the added value of ML in clinical risk stratification.

## 8. Multimodality Imaging

Recent evidence confirms that fusing complementary imaging sources and analytic techniques markedly enhances the detection and risk-stratification of coronary atherosclerosis. The key findings derived from the examined methods are presented in Table 6.

Bae et al. [67] trained an artificial neural network on >40,000 IVUS–OCT co-registered frames; the model predicted OCT-defined TCFA with an 82% accuracy and an AUC of 0.82, outperforming classical support vector machine and naïve Bayes classifiers. This work established a blueprint for “virtual OCT”, whereby low-resolution ultrasound is enriched with texture-based learning to recover cap-level detail.

Using a different fusion paradigm, Bajaj et al. compared machine learning plaque segmentation on chemically sensitive NIRS-IVUS with a parallel OCT pipeline against histology. In 131 NIRS-IVUS/184 OCT frame pairs from human cadaveric hearts, NIRS-IVUS-ML achieved higher concordance with histology for plaque area (CCC 0.88) and overall composition accuracy (83%) than OCT-ML (72%), underscoring the additive value of spectroscopic data [68].

Huang et al. focused on calcification, applying a deep learning module (OctPlus) to OCT pullbacks obtained five years after bio-resorbable scaffold implantation and cross-validating results with OCT optical property mapping, greyscale IVUS, IVUS virtual histology, and echogenicity. The multimodal check showed substantial agreement (weighted κ 0.77) and calcium-arc ICC up to 0.81, with an overall slice-level concordance of 90–93%, illustrating how light- and sound-based traits can be harmonized to overcome modality-specific artifacts [59].

Beyond static phenotyping, Lv et al. [69] combined IVUS + OCT geometries with biomechanical finite-element modelling to compute plaque stress/strain indices and used a random-forest classifier to predict their evolution on follow-up. The best multimodal predictor (plaque and cap morphology plus mechanical stress/strain) reached 90.3% accuracy and an AUC of 0.877 for the cap thickness vulnerability index, a ~9% improvement over morphology alone. These data suggest that mechanical cues captured only when high-resolution OCT is merged with deep IVUS penetration are crucial for longitudinal risk modelling.

Han et al. [70] demonstrated a non-invasive route to multimodality: a CNN trained jointly on CCTA and matched OCT frames accurately classified calcified, non-calcified, and mixed plaques (McNemar *p*  >  0.68 vs. OCT) and detected ≥50% stenosis with an AUC of 0.98—clearly superior to conventional CCTA (AUC 0.76). AI-derived CT-FFR also correlated strongly with quantitative flow reserve (ICC 0.745), linking anatomical and functional assessment in a single workflow.

Finally, the prospective REVEALPLAQUE study further strengthens the fusion concept: Narula et al. found excellent agreement between AI-enabled CCTA plaque volumes and IVUS across 432 lesions (TPV r = 0.91; slope = 0.99), endorsing deep learning CCTA as a non-invasive surrogate for intravascular imaging [71].

Collectively, these studies confirm that multimodal fusion—whether by co-registering invasive images, adding spectroscopic channels, coupling imaging to biomechanics, or integrating non-invasive CT with intravascular ground truths—consistently raises diagnostic accuracy, enriches tissue characterization, and enables personalized prognostication.

## 9. Discussion

A thorough analysis of the collected data confirms that each method—OCT, IVUS, and CCTA—presents specific advantages but also structural limitations that reduce their effectiveness when used in isolation [40,44,45].

### 9.1. Pros and Cons of Each Modality

Optical coherence tomography (OCT), intravascular ultrasound (IVUS), and coronary computed-tomography angiography (CCTA) interrogate the same atherosclerotic substrate through fundamentally different physical principles, and those principles explain both their celebrated strengths and their nagging blind spots.

OCT exploits near-infrared light; the short wavelength produces an axial resolution of 10–20 µm, exquisitely suited for measuring fibrous cap thickness below the histological threshold of 75 µm and visualizing macrophage clusters, micro-channels, and surface thrombus [40]. The price for this microscopic view is a penetration depth of barely 1–2 mm, shadowing from large calcium plates, and the need for contrast flush to displace blood—factors that extend procedure time, raise cost and limit assessment of the deeper plaque core.

IVUS, in contrast, fires 20–60 MHz soundwaves through the vessel wall; resolution drops to 100–200 µm, yet the acoustic beam crosses the full arterial thickness, enabling robust quantification of plaque burden and positive remodelling—features repeatedly linked to clinical events [44]. However, ultrasound cannot resolve thin caps, struggles to discriminate macrophage-rich regions, and is plagued by acoustic shadowing behind dense calcium.

CCTA delivers a panoramic, catheter-free survey of the entire coronary tree. Although its spatial resolution (~1 mm) is an order of magnitude coarser than OCT, it detects vulnerability indirectly through surrogate signs such as napkin-ring morphology, low-attenuation plaque, and positive remodelling [41,45]. Motion artifacts and blooming from calcium can blur boundaries, and inflammatory activity remains beyond native CT’s reach.

### 9.2. Clinical Consequences of Technical Constraints

These technical ceilings translate into tangible clinical pitfalls. The shallow OCT field can underestimate the true atherosclerotic burden and miss positive remodelling. Conversely, the coarse IVUS resolution can miss the thin fibrous cap—one of the strongest histological predictors of rupture—and its insensitivity to macrophage infiltration may reduce prognostic power. For CCTA, indirect surrogates reduce specificity: napkin-ring sign and low-attenuation plaque confer odds ratios of 16.9 and 11.2, respectively, for thin-cap fibroatheroma and macrophage infiltration, yet individual lesions remain subject to false positives due to beam hardening, partial volume effects, and hemodynamic factors [41].

### 9.3. Emerging Solutions—Beyond Single-Modality Silos

Three converging strategies can mitigate these limitations.

- Advanced deep learning architectures. Spatial–temporal encoder–decoders harness frame-to-frame continuity along OCT pullbacks, pushing Dice coefficients for calcification segmentation above 0.75 and F1-scores towards human concordance [48]. Transformer networks, inherently adept at long-range dependencies, now outperform classic CNNs for erosion detection (AUC 0.94 vs. 0.85) [58]. On CCTA, networks such as PlaqueNet and nnU-Net reach sensitivities > 0.90 for vulnerable plaque identification [56,63].
- Radiomics and explainable AI. High-order texture and shape descriptors extracted from CCTA provide hazard ratios around 2.0 for three-year MACE, eclipsing conventional stenosis metrics [46]. Saliency mapping and Bayesian uncertainty estimates are increasingly embedded in pipelines, granting clinicians a transparent window into model reasoning and confidence intervals—an essential step for regulatory endorsement [9,10,11,12].
- Multimodal fusion. Multimodal fusion is progressively redefining coronary plaque assessment by combining the complementary strengths of intravascular OCT, IVUS, CCTA, and spectroscopic imaging. Early demonstrations with co-registered IVUS–OCT datasets showed that deep learning fusion networks can surpass conventional classifiers in identifying fragile plaque features, largely by balancing OCT’s axial resolution with IVUS’s deeper penetration [67]. The addition of near-infrared spectroscopy brings chemical specificity that further refines lipid/necrotic core discrimination [68], while cross-domain frameworks such as OctPlus highlight how concurrent light–ultrasound inputs reduce modality-specific artifacts and improve annotation reliability [59]. Beyond morphology, the coupling of fused images with finite-element biomechanics has yielded more nuanced risk stratification, emphasizing the role of local wall stress in cap failure pathways [69]. Importantly, CCTA-based fusion models that integrate CT-derived FFR now approach invasive standards for plaque typing and physiological assessment, signalling the possibility of a truly non-invasive, single-session pathway from detection to therapy planning [70].

### 9.4. From Proof-of-Concept to Bedside—An Operational Roadmap

Successful clinical translation demands a structured workflow that respects both technical nuance and patient flow. This requirement forms part of the broader historical transition from qualitative to quantitative analyses in medicine, a process now being accelerated by artificial intelligence [72].

- Step 1: Standardized acquisition. Harmonized OCT flush protocols, constant-speed IVUS pullbacks, and ECG-gated, kernel-matched CCTA acquisitions minimize cross-site variance [40,44,45].
- Step 2: Curated, multicentre repositories. Datasets enriched with complementary ground-truth—histology for cap thickness, OCT for surface detail, IVUS for deep components—offer balanced training and unbiased validation.
- Step 3: Hierarchical multimodal networks. A primary segmentation backbone (e.g., 3D U-Net) feeds modality-specific heads that learn domain-unique cues, converging in a fusion layer that adjudicates discordances; early studies report F1-scores up to 0.96 [48,52,58,60,62,64,65].
- Step 4: External prospective validation. Embedding algorithms in multi-vendor, outcome-driven registries will clarify incremental prognostic value over SYNTAX, CAD-RADS, or calcium scoring.
- Step 5: Point-of-care deployment. Integration within angiography consoles or cloud-based CT workstations should deliver a structured risk report in under a minute, aligning with catheterization lab workflow and outpatient CT reporting schedules [56,63].

### 9.5. Clinical Implications in Light of the PREVENT Trial

The recently published multicentre PREVENT randomized trial [73] enrolled 1606 patients with non-flow-limiting but imaging-defined vulnerable plaques (≥2 of small MLA < 4 mm^2^, plaque burden > 70%, maxLCBI_4_ mm > 315, or OCT-defined TCFA) and demonstrated that preventive PCI on such lesions, compared to optimal medical therapy alone, reduced the 2-year composite of cardiac death, target-vessel MI, ischemia-driven revascularisation, and unstable angina from 3.4% to 0.4% (absolute risk reduction 3.0%; *p* = 0.0003), with sustained benefits at 7 years.

These findings provide first-in-class evidence that accurate plaque phenotyping can translate into tangible outcome improvement and may prompt an expansion of PCI indications beyond hemodynamically significant lesions. Automated plaque characterization models reviewed herein—delivering near-expert segmentation and feature extraction across OCT, IVUS and CCTA—could critically enable large-scale identification of treatable vulnerable plaques, lowering operator dependency and analysis time while facilitating prospective, imaging-guided preventive strategies.

## 10. Limitations

The present narrative review synthesizes the rapidly expanding literature on ML–ML-assisted coronary plaque assessment across OCT, IVUS, and CCTA; nevertheless, several constraints must be acknowledged. First, the field is dominated by small, single-centre or vendor-specific datasets, which limits the generalisability of reported performance metrics and increases the risk of selection bias. Second, methodological heterogeneity—encompassing divergent image acquisition protocols, ground truth definitions, and evaluation endpoints—hampers direct comparison between studies and precludes meta-analytic pooling. Third, only a minority of models undergo external or prospective validation, and virtually none are tested for their incremental value over established clinical risk scores in outcome-driven cohorts. Fourth, most published architectures remain “black boxes”; the absence of explainability, transparent code, and open data repositories raises reproducibility concerns. Finally, ethical and regulatory considerations warrant explicit mention. The performance of ML models is tightly coupled to demographic and acquisition biases; uncorrected, these may widen healthcare disparities. Current frameworks (e.g., EU AI Act and FDA SaMD) demand evidence of fairness, post-market surveillance, and clear human oversight. Embedding explainable AI modules (e.g., saliency maps) and reporting calibrated uncertainty estimates are therefore essential to secure regulatory clearance and clinician trust.

## 11. Future Directions and Clinical Implications

To translate AI-enhanced plaque analysis from bench to bedside, future investigations should prioritize the creation of large, multicentre, publicly accessible repositories with harmonized imaging protocols and standardized reference annotations. Multimodal frameworks that fuse complementary information from OCT, IVUS, and CCTA—potentially enriched with radiomics, hemodynamic modelling, and clinical covariates—are poised to deliver more robust, patient-specific risk stratification tools. Equally important will be the integration of explainable AI techniques (e.g., saliency mapping and feature attribution) and uncertainty quantification, enabling clinicians to trust and audit automated outputs. Prospective, outcome-oriented trials—ideally embedded within interventional or preventive pathways—are essential to demonstrate that AI-driven characterization of vulnerable plaque can refine therapeutic decision-making, guide device selection, and ultimately improve hard cardiovascular end points.

## 12. Conclusions

ML applications in coronary imaging have progressed from proof-of-concept segmentation tasks to sophisticated, near-real-time systems capable of delineating high-risk morphological features with expert-level accuracy. When coupled with emerging multimodal and radiomic approaches, these algorithms offer a unique opportunity to transform plaque evaluation from a subjective, labour-intensive exercise into a rapid, reproducible and comprehensive biomarker of patient-level risk. Notwithstanding current limitations, the convergence of open data initiatives, explainable model design, and rigorously conducted clinical trials is likely to accelerate the incorporation of AI-assisted plaque assessment into the diagnostic and prognostic armamentarium endorsed by future iterations of cardiovascular guidelines.

## Figures and Tables

**Table 1:** Comparison between the traditional AHA classification and the morphological scheme proposed by Virmani et al. for coronary atherosclerotic lesions.

AHA Classification	Virmani et al. Classification	Key Differences
Type I—Initial lesion	Intimal xanthoma	Both describe early foam cell accumulation. Virmani uses a standard pathological term.
Type II—Fatty streak	Intimal xanthoma	Virmani emphasizes its potential regression and uses more precise terminology.
Type III—Intermediate lesion	Pathological intimal thickening	Describes lesions with extracellular lipids but no necrosis; emphasizes morphologic criteria.
Type IV—Atheroma	Fibrous cap atheroma	Focuses on the presence of a necrotic core and a well-formed fibrous cap.
Type V (Va, Vb, Vc)—Fibroatheroma/calcified/fibrotic	Fibrous cap atheroma/fibrocalcific plaque	Virmani simplifies subdivisions with descriptive terms based on morphology and stability.
Type VI—Complicated lesion	Plaque rupture/erosion/calcified nodule	Virmani differentiates mechanisms leading to thrombosis instead of grouping all under “complicated”.

**Table 2:** Comparison of advantages, disadvantages, and adaptability of the main machine learning and deep learning models.

Model/Family	Advantages	Disadvantages	Adaptability
Random Forest/SVM	Easy to interpret; train quickly on small datasets	Limited performance on raw imaging; needs feature engineering	High—retune hyperparameters only
2D-CNN	High accuracy on single-frame OCT/IVUS; mature libraries	Requires datasets > 10^3^ images; needs GPU	Medium—fine-tune with a small site-specific set
3D/2.5D CNN (U-Net, SegNet)	Ensures spatial consistency; Dice ≈ 0.9 for segmentation	High RAM usage; overfitting risk on small cohorts	Good after pre-training on multicentre databases
Transformer	Captures long-range context; AUC > 0.9 for erosion detection	GPU/TPU intensive; needs > 10^4^ examples	Emerging—promising with transfer learning
GAN	Powerful for data augmentation; real-time IVUS inference	Training instability; sensitive hyper-parameter tuning	High for augmenting new cohorts
Ensemble/Hybrid	Improves multicentre robustness	Complex pipeline; harder to debug	High—retrain component models only

Key strengths, limitations/data requirements, and expected ease of cross-centre transferability for six algorithmic families—Random Forest/SVM, 2D-CNN, 3D/2.5D-CNN (U-Net, SegNet), transformer, GAN, and ensemble/hybrid approaches—providing a concise reference for selecting the most suitable architecture in intravascular imaging studies.

**Table 3:** Comparison of OCT, IVUS, and CT imaging capabilities in the evaluation of coronary artery atherosclerotic plaque features, providing an overview of their respective roles.

Feature Type	OCT	IVUS	CCTA
Thin-cap fibroatheroma	High resolution (10–20 μm) for accurate measurement	Limited resolution (100–200 μm) for cap thickness	Indirect detection through napkin-ring sign
Lipid content	High accuracy in detecting lipid-rich plaques	Moderate accuracy, improved with VH-IVUS	Low attenuation plaque as a surrogate marker
Fibrous cap thickness	Precise measurement capability	Limited by resolution	Not directly measurable
Macrophage infiltration	Capable of detecting and quantifying	Limited capability	Not directly detectable
Positive remodelling	Detectable, but limited field of view	Excellent capability	Detectable and quantifiable
Spotty calcification	High-resolution detection	Detectable	Detectable, size-limited definition
Microchannels	Unique capability to detect	No mention found	No mention found

**Table 4:** Segmentation performance analysis.

Authors	Year	*N*	Imaging Modality	Variables	ML Models	Performance Metrics	Validation Method	Key Results
Zhang C., et al. [47]	2019	5	OCT, IVUS	Lumen border, plaque components (lipid, fibrous, background)	CNN (U-Net), SVM	Classification accuracy (CNN: 95.8%, SVM: 71.9%)	11-fold cross-validation	CNN provided superior segmentation performance; significantly reduced manual effort
Li C., et al. [48]	2022	45 *	IVOCT	Calcification area, depth, angle, thickness, volume, calcification score	2.5D U-Net, DenseNet	Dice coefficient (0.756 ± 0.222), F1-score (0.883 ± 0.008), Precision (0.964 ± 0.002)	Comparison with human-level inter-observer agreement	Accurate and efficient segmentation and classification of calcification with near-human agreement
Lee J., et al. [49]	2020	68 †	IVOCT	Major calcification lesions	3D CNN + SegNet	F1-score (0.781), Sensitivity (86.2%), Precision (75.8%)	Comparison with one-step approach; reproducibility tests	Two-step deep learning improved performance and reproducibility, enabling real-time planning
Matsumura M., et al. [50]	2023	326 ‡	IVUS	Lumen and vessel dimensions, stent area, balloon size	U-Net	IoU (0.92–0.94), DSC (0.96–0.97), Correlation (0.991–0.993)	Expert comparison, independent test set	High correlation with experts; 92.4% agreement on balloon size, >85% agreement on lumen/stent area
Cui H., et al. [51]	2020	3 §	IVUS	Lumen area and contour	Gradient Boosting with handcrafted features	Jaccard similarity (96.8%), Mean error distance (0.55)	IVUS Challenge benchmark + manual annotation comparison	Outperformed other ML methods; highly accurate lumen segmentation with minimal error
Bajaj R., et al. [52]	2021	65 ¶	IVUS (NIRS-IVUS)	EEM and lumen borders, plaque area	Pix2Pix GAN + ResNet	Mean difference ≤ 0.23 mm^2^; DSC (0.96–0.98), IOU (0.92–0.96)	Compared to two expert analysts; Williams Index (0.75–1.06)	Real-time DL segmentation as accurate as experts; robust even in complex images
Dong C., et al. [53]	2023	119	CCTA	Coronary artery anatomy	CAS-Net (multi-attention, multi-scale 3D network)	DSC: improvement of at least 4% over U-Net3D	Comparison with three public datasets + self-collected data	Outperformed 14 state-of-the-art methods; strong generalization and accuracy
Serrano-Antón B., et al. [54]	2025	32	CCTA	Coronary arteries	Ward Clustering (3Axis, Perp), VGG-19, ResNet-50, EfficientNet-b2, U-Net++, 3D U-Net, Swin UNETR	Dice (0.88 test, 0.83 lesion), IoU, Precision, Recall	Manual annotation comparison on 10 test and 22 lesion cases	3Axis clustering comparable to advanced DL models; Swin UNETR best Dice (0.8978) but more complex
Nannini G., et al. [55]	2024	324 **	CCTA	Coronary artery segmentation, CAC, tortuosity	2.5D U-Net + 3D U-Net cascaded	DSC (0.895), Surface distance (0.47 mm), Precision (93.5%)	Manual annotations, test set inference	Accurate segmentation of stenotic regions, robust CAC and CorT extraction
Wang L., et al. [56]	2024	NS ††	CCTA	Coronary plaques	PlaqueNet (AResNet + DASPP-BICECA + BINet)	IoU: 87.37%, Dice: 93.26%, Mean Dice: 96.63%	Comparison with 3 other models	Highest performance among tested models; sensitive and precise plaque detection

* 13,844 images from 45 pullbacks; † 8231 clinical images + 4320 cadaveric images; ‡ 234 (training/validation) + 92 (test); § 77 manually labeled frames from the public IVUS Challenge 2011 dataset + 1,450 frames from 3 real-world clinical pullbacks; ¶ 197 IVUS sequences; ** 281 training + 43 test; †† NS: Not specified. OCT: Optical Coherence Tomography; IVUS: Intravascular Ultrasound; IVOCT: Intravascular OCT; CCTA: Coronary Computed Tomography Angiography; CNN: Convolutional Neural Network; SVM: Support Vector Machine; CAC: Coronary Artery Calcium; CorT: Coronary Tortuosity; DSC: Dice Similarity Coefficient; IoU: Intersection over Union; EEM: External Elastic Membrane; ML: Machine Learning; DL: Deep Learning; GAN: Generative Adversarial Network.

**Table 5:** Plaque detection performance.

Authors	Year	*N*	Imaging Modality	Variables	ML Models	Performance Metrics	Validation Method	Key Results
He C., et al. [57]	2020	24 *	OCT	Calcified vs. non-calcified plaque	ResNet-3D, ResNet-2D	F1-score (up to 96%)	10 runs with cross-validation, split into train/validation/test	ResNet-3D outperformed ResNet-2D; 3D model achieved up to 96% F1-score
Park J., et al. [58]	2022	873 †	OCT	Plaque erosion	CNN, transformer	AUC, Sensitivity, Specificity, PPV, NPV	Internal and external validation	Transformer model outperformed CNN (AUC 0.94 vs. 0.85 externally)
Li C., et al. [48]	2022	45 ‡	IVOCT	Calcification segmentation and classification	Spatial–temporal encoder–decoder + DenseNet	Dice coefficient, Precision, F1-score	Comparative study vs. other methods	F1-score improved from 0.791 to 0.883; Dice from 0.615 to 0.756
Huang J., et al. [59]	2022	15 ¶	OCT + IVUS	Calcified plaques (pure, hybrid)	OCT-DL (OctPlus), optical and ultrasound validation	Kappa statistics, ICC for arc measurements	Cross-validation with IVUS and optical signals	Substantial agreement (kappa > 0.69); ICC up to 0.81
Cho H., et al. [60]	2021	598 ††	IVUS	Attenuated plaque, calcified plaque	EfficientNet (ensemble of 5 models)	Dice, Accuracy, Sensitivity, Specificity	5-fold cross-validation, test on separate set	Frame-level accuracy: 93% (attenuation), 96% (calcification); Dice up to 0.84
Li YC., et al. [61]	2021	18 ‡‡	IVUS	Media–adventitia border, lumen, calcified plaque	Cascaded modified U-Nets	Dice, Precision, Sensitivity, Specificity, AP	Leave-one-subject-out cross-validation	Calcification AP up to 0.73; Dice for media/lumen > 0.90; superior to VH-IVUS in artifact conditions
Chen Q.., et al. [62]	2023	933 §§	CCTA + IVUS (reference)	Vulnerable plaques, MACE risk	XGBoost (radiomic signature)	AUC, Hazard ratio	Internal and external test sets, Cox regression for MACE	AUC: 0.81–0.77 (train to external test); HR for MACE: 2.01; validated in multicenter cohort
Kim D. [63]	2023	3747 ***	CCTA	Stenosis, calcification, vulnerable plaques	2D nnU-Net segmentation	Correlation (QSI vs. QCA), Sensitivity, Specificity	Expert annotation and independent test	Sensitivity 0.929, Specificity 0.910 for VP detection
Buckler AJ., et al. [64]	2023	53 ‡‡‡	CCTA + Histopathology	Plaque phenotype (stable/unstable/minimal)	ML classifier with ResNet-18 architecture	AUC, Kappa	Radiologic-pathologic validation	AUC: 0.97 (unstable), 0.95 (stable); Kappa = 0.82
Al’Aref SJ., et al. [65]	2020	468 §§§	CCTA + ICA	Culprit lesion prediction	Boosted ensemble (XGBoost)	AUC	10-fold cross-validation, test set	AUC: 0.77 (ML) vs. 0.60–0.67 (traditional); specificity: 89.3%
Yang S., et al. [66]	2021	1013 (vessels)	CCTA		Boruta + hierarchical clustering	HR	Prospective registry with FFR	HR 5.43 when ≥4/6 features

* 18 training/validation, 6 test; † 581 training/validation, 292 external validation; ‡ 45 pullbacks (13,844 images); ¶ 72 cross-sections; †† 498 training, 100 test; ‡‡ 713 images; §§ 225 development, 708 prognostic validation; *** 2978 training/validation, 769 test; ‡‡‡ 30 derivation, 23 validation; §§§ ICONIC study, nested case–control. OCT: Optical Coherence Tomography; IVUS: Intravascular Ultrasound; IVOCT: Intravascular OCT; CCTA: Coronary Computed Tomography Angiography; DSA: Digital Subtraction Angiography; ICA: Invasive Coronary Angiography; CNN: Convolutional Neural Network; ML: Machine Learning; DL: Deep Learning; AUC: Area Under Curve; PPV: Positive Predictive Value; NPV: Negative Predictive Value; ICC: Intraclass Correlation Coefficient; AP: Average Precision; MACE: Major Adverse Cardiovascular Event; VH-IVUS: Virtual Histology IVUS; CAS: Coronary Artery Stenosis; VP: Vulnerable Plaque; QSI: Quantitative Stenosis Index; QCA: Quantitative Coronary Angiography; HR: Hazard Ratio.

**Table 6:** Multimodality model performance.

Author	Year	*N*	Imaging Modality	Clinical/Technical End-Point	ML Models	Performance Metrics	Validation Method
Bae Y., et al. [67]	2019	517 lesions/40 908 frames	IVUS → OCT (label)	Prediction of OCT-TCFA	ANN, SVM, NB	Accuracy 82%, AUC 0.82	5-fold CV + independent test set
Bajaj R., et al. [68]	2025	131 NIRS-IVUS + 184 OCT/histology pairs	NIRS-IVUS + OCT + histology	Plaque component quantification	Dedicated ML classifiers	Accuracy 83% (NIRS-IVUS), CCC 0.88	Cadaveric histology reference
Huang J., et al. [59]	2022	15 pts/72 slices	OCT-DL + IVUS + optical props.	Calcified plaque detection	OctPlus DL	κ 0.77; ICC 0.81; agreement > 90%	Cross-validation with IVUS and optics
Lv R., et al. [69]	2023	10 pts/228 slices	IVUS + OCT + biomechanics	Prediction of vulnerability index change	Random Forest	Accuracy 90.3%, AUC 0.877	5-fold CV, baseline vs. follow-up
Han J., et al. [70]	2025	100 pts	CCTA + OCT (+QFR)	Plaque type and CT-FFR	Multi-stage CNN	AUC 0.98 (>50% stenosis); ICC 0.91 (MLD)	Hold-out test; comparison with manual OCT and QFR
Narula J., et al. [71]	2024	237 pts	CCTA ↔ IVUS	Plaque volume agreement	AI-QCPA DL	TPV r 0.91; slope 0.99	Prospective, 15-centre

ANN: artificial neural network; NB: naïve Bayes; CCC: concordance correlation coefficient; κ: kappa; ICC: intraclass correlation coefficient; CV: cross-validation.

## Data Availability

No new data were created or analyzed in this study. Data sharing is not applicable to this article.

## Supplementary Materials

The following supporting information can be downloaded at https://www.mdpi.com/article/10.3390/diagnostics15141822/s1, Table S1: Detailed database search queries.

## Abbreviations

The following abbreviations are used in this manuscript:

OCT	Optical coherence tomography
IVUS	Intravascular ultrasound
CCTA	Coronary computed tomography angiography
CAD	Coronary artery disease
ML	Machine learning
DL	Deep learning
AI	Artificial intelligence
HRP	High-risk plaque
MACE	Major adverse cardiovascular event(s)
