# Supplementary material for: Machine Learning for Coronary Plaque Characterization: A Multimodal Review of OCT, IVUS, and CCTA
Source: Diagnostics (Basel). 2025 Jul 19;15(14):1822. doi: 10.3390/diagnostics15141822 (PMC12293362; doi:10.3390/diagnostics15141822)
Supplement: Supplementary file 1 [file diagnostics-15-01822-s001.zip › diagnostics-3674434-supplementary.pdf]

Table S1. Detailed database search queries.

Database	Search query
PubMed	((coronary OR cardiac OR heart OR cardiovascular) AND (atherosclerosis OR plaque) AND (OCT OR "optical coherence tomography" OR IVUS OR "intravascular ultrasound" OR CCTA OR CT OR "computed tomography") AND ("artificial intelligence" OR AI OR "machine learning" OR ML OR "deep learning" OR algorithm)) AND ("2019"[Date-Publication] : "2025"[Date-Publication])
Scopus	TITLE-ABS-KEY((coronary OR cardiac OR heart OR cardiovascular) AND (atherosclerosis OR plaque) AND (OCT OR "optical coherence tomography" OR IVUS OR "intravascular ultrasound" OR CCTA OR CT OR "computed tomography") AND ("artificial intelligence" OR AI OR "machine learning" OR ML OR "deep learning" OR algorithm)) AND PUBYEAR > 2018 AND PUBYEAR < 2026
Embase	((coronary OR cardiac OR heart OR cardiovascular) AND (atherosclerosis OR plaque) AND (OCT OR 'optical coherence tomography' OR IVUS OR 'intravascular ultrasound' OR CCTA OR CT OR 'computed tomography') AND ('artificial intelligence' OR AI OR 'machine learning' OR ML OR 'deep learning' OR algorithm)) AND py=2019-2025
Google Scholar	(coronary OR cardiac OR heart OR cardiovascular) (atherosclerosis OR plaque) (OCT OR "optical coherence tomography" OR IVUS OR "intravascular ultrasound" OR CCTA OR CT OR "computed tomography") ("artificial intelligence" OR AI OR "machine learning" OR "deep learning" OR algorithm)
